# Microstructure Evaluation and Mechanical Properties of PMMA/Al_2_O_3_ Nanocomposite Fabricated via Friction Stir Processing

**DOI:** 10.3390/polym18172093

**Published:** 2026-08-28

**Authors:** Reham K. Elsawah, N. S. M. El-Tayeb, Mohamed M. Z. Ahmed, Salem M. Aldosari, Mohamed M. El-Sayed Seleman

**Affiliations:** 1 Faculty of Sustainable Design Engineering, Universities of Canada, Cairo 11835, Egypt; reham.elsawah@uofcanada.edu.eg; 2 Department of Mechanical Engineering, Faculty of Engineering, The British University in Egypt (BUE), Suez Desert Road, Al-Shorouk, Cairo 11837, Egypt; nabil.eltayeb@bue.edu.eg; 3 Mechanical Engineering Department, College of Engineering at Al Kharj, Prince Sattam bin Abdulaziz University, Al Kharj 11942, Saudi Arabia; moh.ahmed@psau.edu.sa; 4 Department of Metallurgical and Materials Engineering, Faculty of Engineering, Atatürk University, Erzurum 25240, Türkiye; 5 Material Science Research Institute, King Abdulaziz City for Science and Technology (KACST), Riyadh 11442, Saudi Arabia; 6 Department of Metallurgical and Materials Engineering, Faculty of Petroleum and Mining Engineering, Suez University, Suez 43512, Egypt; mohamed.elnagar@suezuniv.edu.eg

**Keywords:** friction stir processing, polymer matrix nanocomposite, mechanical properties, microstructure

## Abstract

This study aimed to develop polymer matrix nanocomposites reinforced with Al_2_O_3_ nanoparticles to enhance the mechanical properties of PMMA. The composite was fabricated via friction stir processing (FSP) to ensure the homogenous dispersion of Al_2_O_3_ nanoparticles in the polymer. A grid of 5 holes in a 7 × 7 mm^2^ area was made in which the hole diameter was varied from 1.77 mm to 2.28 mm to obtain different volume fractions of reinforcement ranging from 15% to 25%. The holes were made with a depth of 3 mm in a 4 mm-thick PMMA sheets. After packing the Al_2_O_3_ powder in the holes, a 2 mm-thick PMMA sheet was used as a cover to prevent the sputtering of nanoparticles. A number of FSP parameters were examined. The tool rotation rates ranged from 800 to 1200 rpm, traverse speeds of 25 and 50 mm/min, and tool tilts of 1 and 2° were used. A soft paraffin (Vaseline) layer was used on the top surface to prevent severe shoulder friction with the PMMA plate, which caused severe wear and thinning on the surface. For the developed PMMA/Al_2_O_3_ nanocomposites, the surface quality, SEM microstructure, impact energy, and transverse hardness were investigated. Good surface quality and dispersion of nanoparticles were attained by employing adequate processing conditions. The experimental results indicated that as the nanoparticle percentage increased, impact energy, hardness, and tensile strength increased, reaching 2 kJ/m^2^, 14.7 HV, and 52.1 MPa at a nanoparticle concentration of 25%. This means that the polymer ceramic composite’s toughness, hardness, and tensile strength are higher than those of unprocessed PMMA by 66%, 33%, and 23%, respectively.

## 1. Introduction

In the past few decades, composite materials have become prominent due to their exceptional mechanical properties and lightweight composition. The use of these materials is expanding rapidly, and they are finding new markets to infiltrate. The current formulations of composites contain a ratio of reinforcing fibers and matrix with varying volume fractions to enhance their properties. Researchers often employ different fillers to further enhance the engineering applications of these composites, which has proved to be very beneficial [1]. Due to their excellent strength-to-weight ratio, hardness, wear resistance, mechanical properties, and relatively low cost, polymer-based composites are used in a wide range of engineering and industrial applications. These applications range from the automotive industry, military, marine technology, and aeronautics to agricultural and biomedical applications [2,3,4,5]. One of the newly introduced techniques for composite fabrication is friction stir processing, where this processing technique provides microstructure modification and grain refinement [6]. Although a few studies have concluded that using this method improved the tensile strength, hardness, and impact energy of polymer-based composites [7,8,9,10,11], some research has reported that friction stir processing destroys the molecular chain, degrades the polymer, changes its molecular weight, and does not necessarily prevent agglomeration, which leads to the deterioration of the polymer’s mechanical properties [12,13]. Thus, processing conditions and particle incorporation techniques are investigated and varied to prevent sputtering of the nanoparticles during processing to ensure uniform processing flow and good nanoparticle dispersion.

Eslami et al. [14] reported that rotational speed, welding speed, and axial force have a significant effect on the processing quality and mechanical properties of the polymer [14], whereby increasing the rotational speed and/or decreasing the TS speed causes frictional heat generation; hence, the maximum temperature is increased. Furthermore, composites with higher transverse speed had more agglomerations due to the inadequate flow of material, as there is more material being processed per 1 min, which leads to uneven melting of the material and poor distribution of the nanoparticles [8,11,14]. Thus, adjusting the processing temperature is challenging since polymers have melting ranges instead of a single melting point, given that their molecule chains are of different lengths. Hence, inhomogeneous distribution, voids, and channels are present due to the suspension of some unmelted solid polymer in the molten polymer matrix, leading to a reduction in mechanical properties. Thus, a decrease in temperature exhibits the agglomeration of nanoparticles and the formation of voids and channels due to the uneven melting of the material, which will cause low mechanical properties of the composite, while a higher temperature shows poor dispersion, which is attributed to the degradation of the material due to overheating. Finally, uneven cooling causes the formation of hard shells, whereas the outer shell cools quicker than the inner part. As the inner layers then cool, the material contracts and pulls away from the shell, which forms large voids that will remarkably reduce the mechanical properties of the fabricated composite [8,11,13].

Surface composites produced through friction stir processing involve incorporating a secondary phase into the matrix using various reinforcement methods [14]. In the hole method, a series of small, closed holes are created at equal distances, filled with powder, compacted, and processed along a specified path [15]. The zigzag hole pattern results in better incorporation of the secondary phase and improved homogeneity [16,17]. In the groove or slot method, one or more grooves are produced across the length of the workpiece, filled with particles, compacted, and then processed using friction stir. The cavities are closed before processing to prevent the scattering of the filled secondary phase [18]. In a different approach, grooves filled with secondary phase are covered by an aluminum plate instead of being capped, and then the plates are clamped facing each other and processed using the friction stir method [19]. The sandwich method involves placing another thin plate on the workpiece surface, cladding it with a parent metal plate, and then processing using friction stir to produce surface composites [20]. The multiple microgroove strategy is more effective than a single macrogroove in achieving better reinforcement distribution and resulting properties [21].

Despite these advances, the reported reinforcement strategies mainly focus on conventional hole, groove, and sandwich configurations. Limited attention has been given to the development of alternative particle incorporation strategies that ensure nanoparticle distribution and prevent surface wear during friction stir processing for PMMA-based nanocomposites. To the best of the authors’ knowledge, the combination of the proposed hole configuration with lubrication-assisted friction stir-processing using soft paraffin has not been previously reported for the fabrication of PMMA/Al_2_O_3_ nanocomposites. Hence, the aim of this work was to fabricate a nano-alumina-reinforced polymethyl methacrylate composite using friction stir processing and to investigate the effect of the nanoparticles and processing on the microstructure, mechanical and tribological properties of the produced nanocomposite. This required investigating the processing conditions and particle incorporation techniques to prevent sputtering of the nanoparticles during the processing and ensure uniform processing flow and good nanoparticle dispersion.

## 2. Experimental Work

### 2.1. Materials

Poly (methyl methacrylate) (PMMA) transparent sheets of 4 mm and 2 mm thickness were supplied by Spiroplastic, Zamalek, Egypt. Nano Alumina particles with a mean size of 9 nm and a purity of 99% were supplied by Nanotech Egypt (Cairo, Egypt).

### 2.2. Specimens Preparation

**Figure 1:**
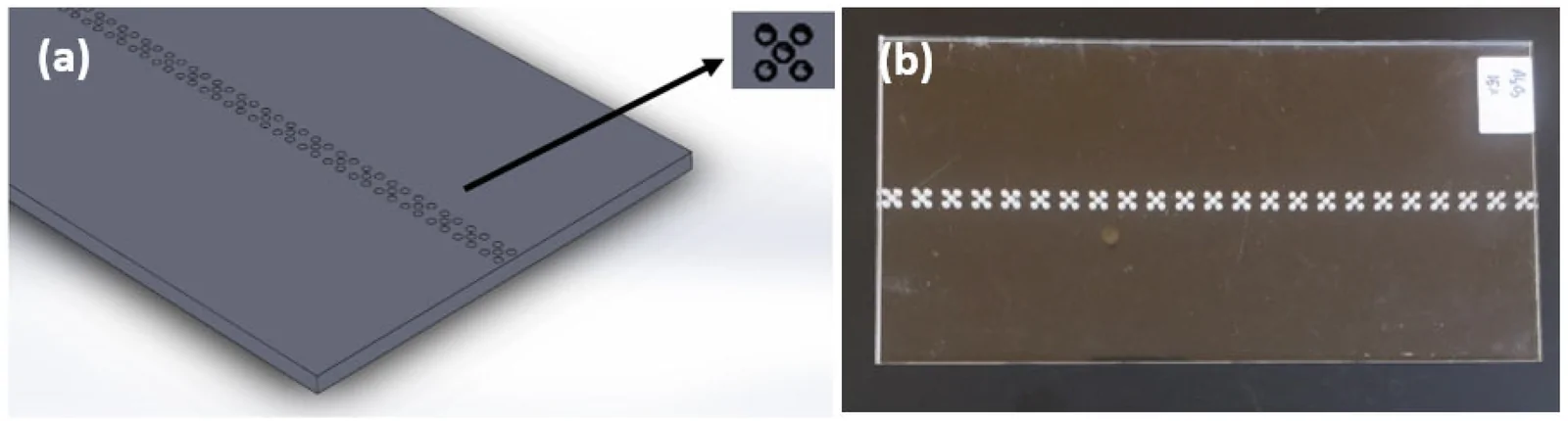
(**a**) PMMA plate drawing showing holes’ configuration; (**b**) PMMA 4 mm plate with holes.

The PMMA sheets were cut into 200 × 100 mm^2^ plates, then holes were drilled in the center of each 4 mm plate with the configuration shown in Figure 1 using a laser cutting machine. This configuration was introduced to ensure homogenous nanoparticle dispersion, as a previous literature review showed that agglomeration was one of the main problems faced during friction stir processing [8,22]. The configuration shown in Figure 1 was repeated every 7 mm, which is the tool pin diameter, whereas the 5 holes were drilled in each 7 × 7 mm area. This configuration was suggested to ensure that there was as much predistribution of nanoparticles as possible, whereas the rotating pin mixed the desired volume percentage of nanoparticles prepositioned in 5 equidistant sites with PMMA. The blind holes had a fixed 3 mm depth, whereas their diameter varied from one specimen to the other according to the volume fraction of nanoparticles required, as shown in Table 1. This was done by calculating the volume of one square of the friction stir processed zone as follows:

(1) Volume of one square of processed zone=length×width×depth

where is the length and width of the square are equal to the diameter of the pin and the depth is fixed to 5 mm, which is the tool plunge depth. For each square, there are five cylindrical blind holes as shown. Hence, to calculate the diameter to achieve the required volume fractions, the following equations were used.

(2) $$\mathrm{Volume}\;\mathrm{of}\;\mathrm{holes}=\frac{5\;\pi D^{2}h}{4}$$

Whereas the height is the plunge depth in the 4 mm plate only, since the blind holes will be in this plate only, Height (*h*) = 5 − 2 = 3 mm.

By substituting the value of the height in the equation, we get:

(3) $$\mathrm{Volume}\;\mathrm{of}\;\mathrm{holes}=\frac{15\;\pi D^{2}}{4}$$

By dividing Equation (3) by Equation (1), the required diameter is obtained to attain the desired nanoparticles’ volume percentage.

Across the FSP literature, ceramics generally improve hardness, strength, and wear when they are well dispersed. Still, the benefit commonly peaks at an optimum loading and then plateaus or reverses once clustering and stress concentration dominate, which is why very high fractions such as above about 25% are generally not favored. Very low fractions often do not justify processing unless the specific particle system is highly efficient at lower loading [23,24,25]. Hence, the investigated range for the nanoparticles is 15, 20, and 25 volume percentage.

### 2.3. Friction Stir Processing

**Figure 2:**
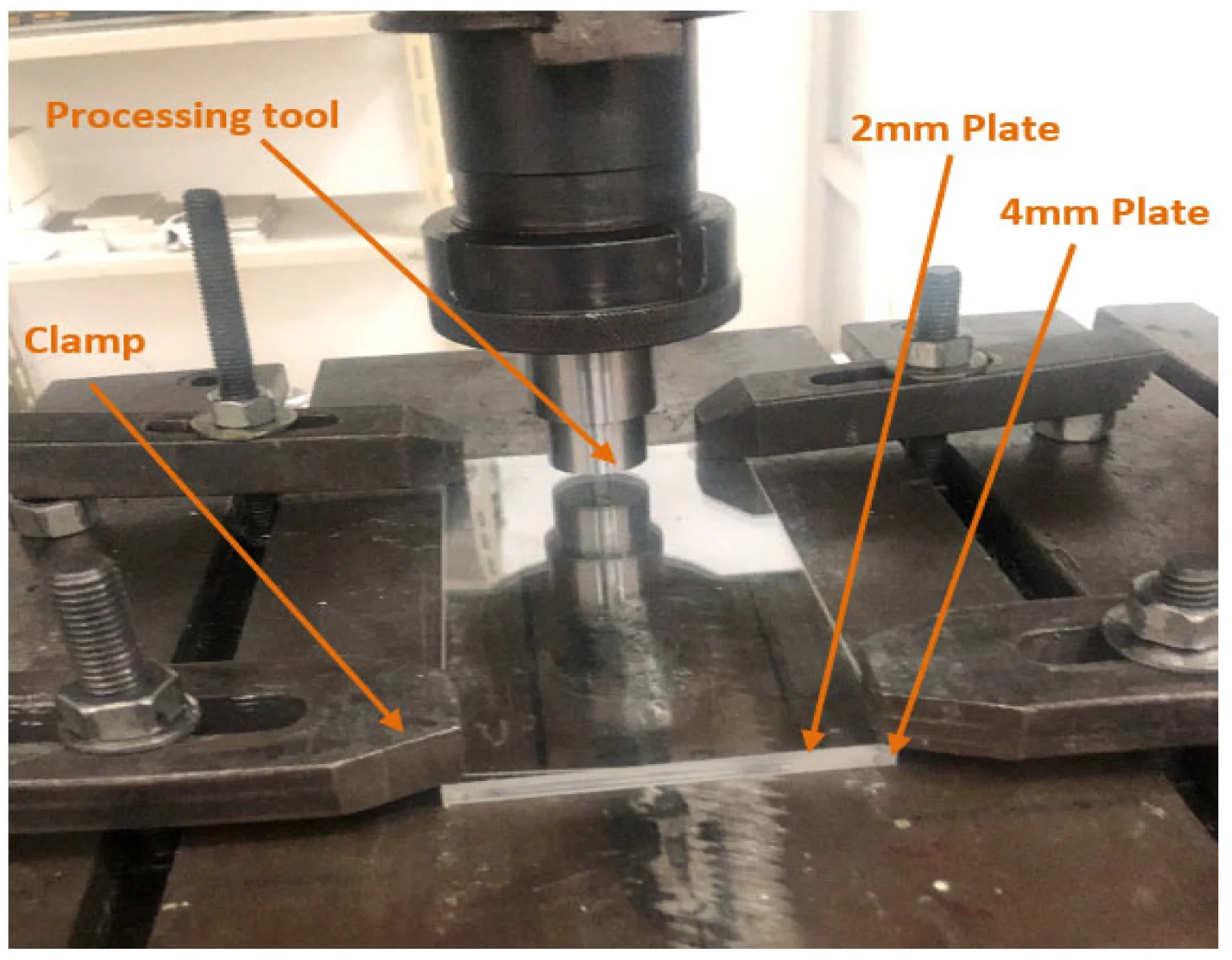
Friction stir processing setup.

After filling the 4 mm plate holes with nanoparticles by mixing Al_2_O_3_ nanoparticles with ethanol to form a slurry, the slurry was incrementally inserted into the pre-drilled 4 mm holes, manually compacted using a fitted pin to maximize packing density, and allowed to dry, the plates were placed on an FSP machine bed to perform friction stir processing using FSP machine (Suez University), where the transverse path of the FSP tool is parallel and coincident with the holes’ configuration. Then the 2 mm PMMA plate was put on top of the 4 mm plate to prevent sputtering of the nanoparticles during friction stir processing. Finally, the plates were clamped to fix them in position, as shown in Figure 2. To determine the processing conditions required to produce the composite, data were gathered from a previous literature review, as shown in Table 2, to establish the ranges successfully employed to process/weld polymer materials.

**Figure 3:**
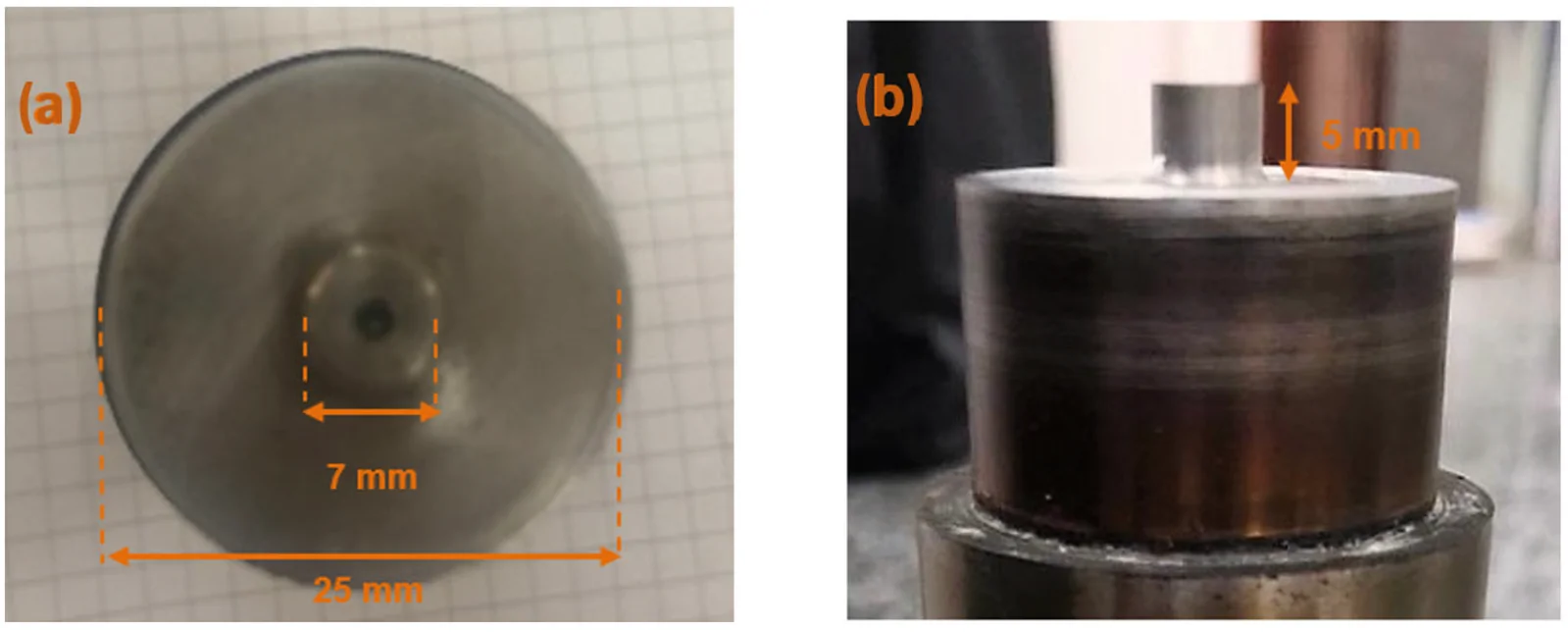
Friction stir processing tool: (**a**) top view; (**b**) front view.

**Figure 4:**
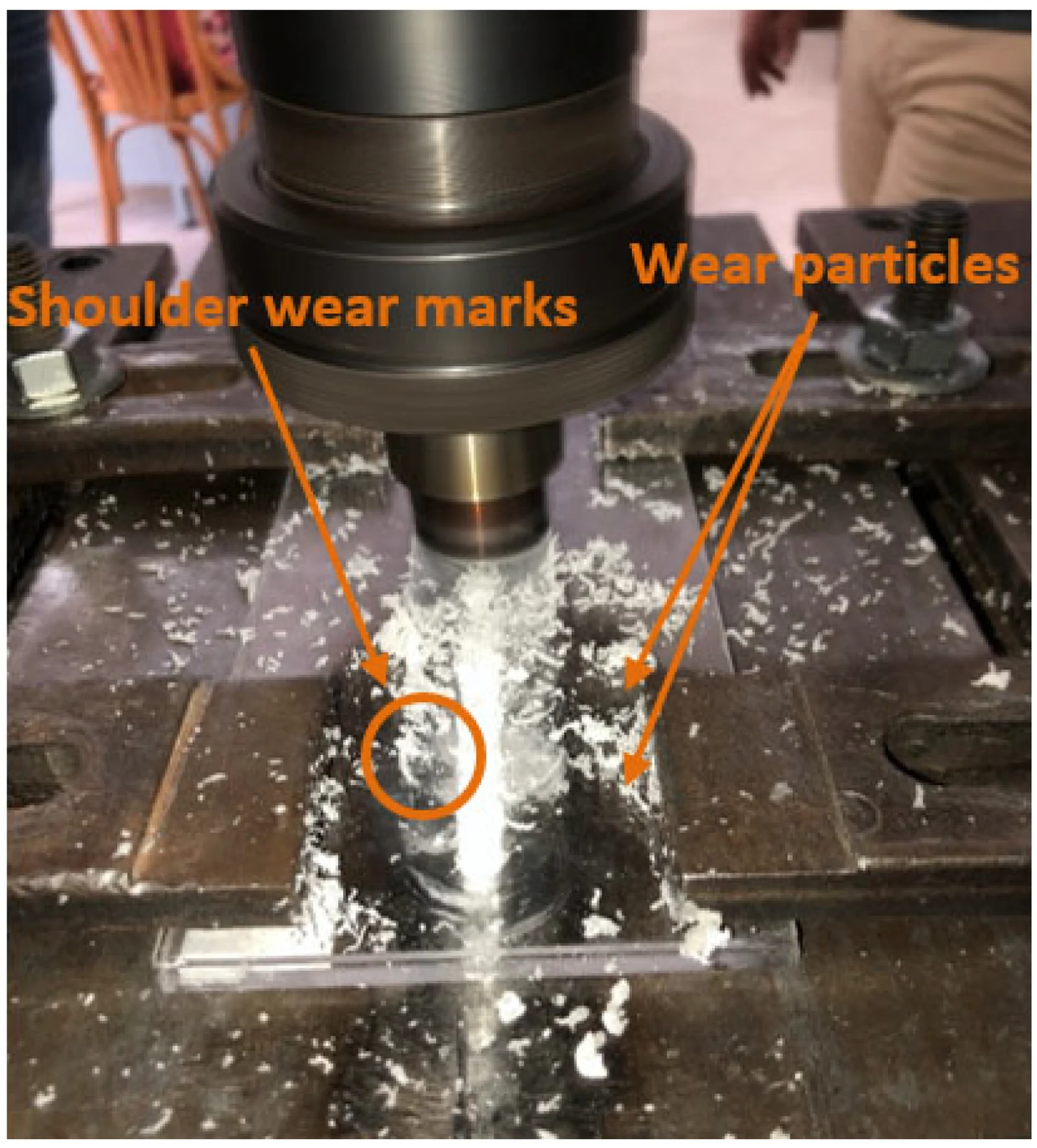
FSP, without using surface lubrication, shows the worn PMMA plate due to stirring.

The tool used for friction stir processing was made from high-speed tool steel (H13) with a cylindrical profiled pin of diameter and length of 7 mm and 5 mm, respectively, and a 25 mm shoulder, as shown in Figure 3. The rotational speed, transverse speed, and tilt angle were varied according to previous literature; the processing parameters investigated are listed in Table 3. However, all the trials and variations in the aforementioned conditions failed to process the PMMA sheet, as they all showed the same problem, which is the wear of the polymer during processing, as shown in Figure 4, where the tool shoulder and pin caused high frictional heat leading to chip removal from the polymer. Hence, to find a solution to the wear problem that is usually faced when friction stir processing is used on a polymer [9], it was suggested to attempt adding a lubricant between the shoulder and the polymer surface during processing to reduce wear.

**Figure 5:**
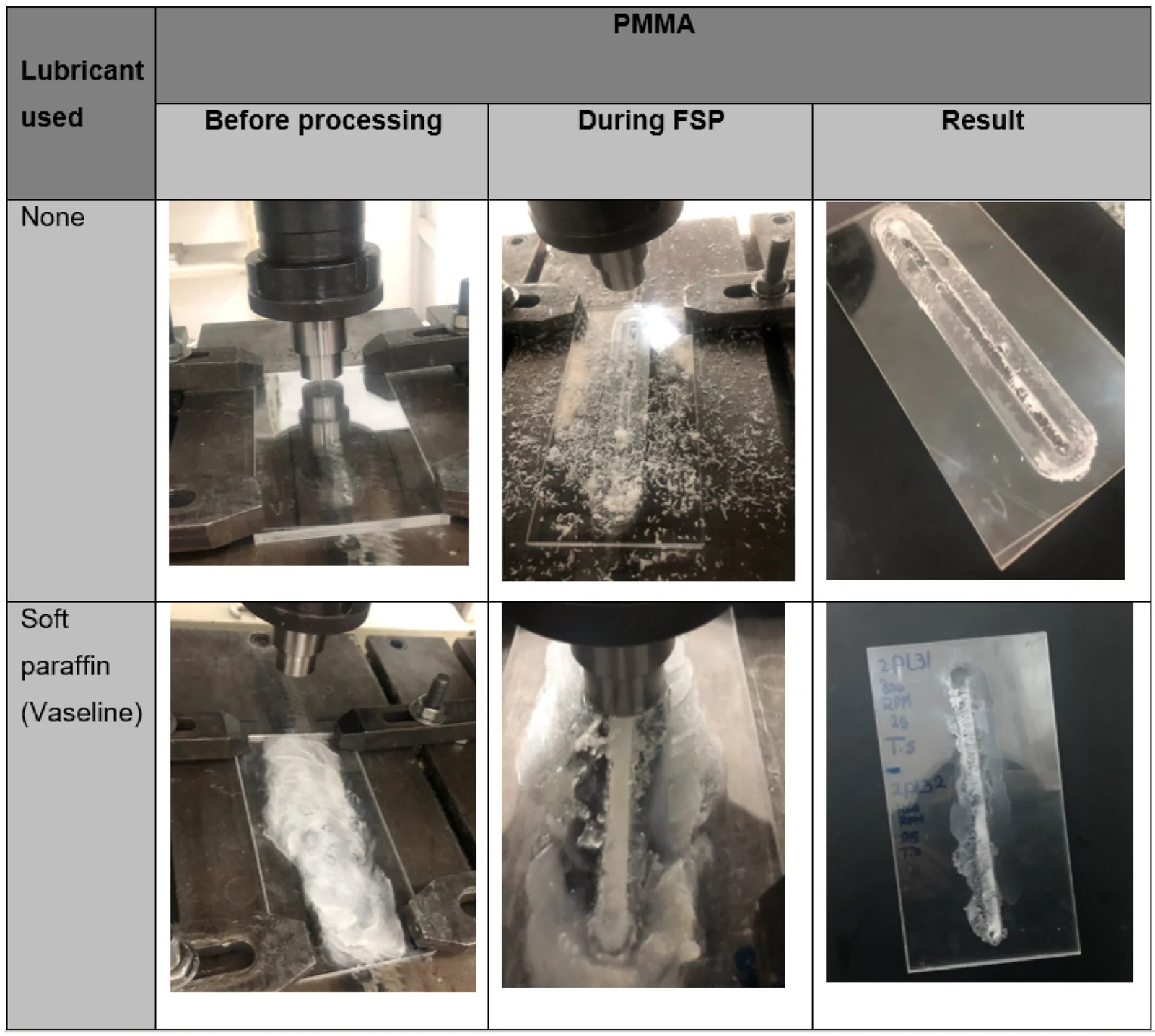
Comparison between friction stir processing of PMMA with and without the addition of soft paraffin.

Attempting the friction stir process on the test specimens, soft paraffin (with the commercial name Vaseline) was applied on top of the 2 mm thick polymer plate prior to friction stir processing to reduce the friction between the shoulder and the plate and preheat the material in order to eliminate wear. The addition of the lubricant eliminated wear and softened the polymer during processing, resulting in successfully processed specimens with no material wear, as shown in Figure 5. During processing, the thin surface paraffin layer melted and was centrifugally displaced outward by the rotating tool, preventing internal entrapment within the stir zone. Post-FSP specimens were thoroughly degreased with high-purity ethanol to remove any remaining surface residue prior to characterization.

**Figure 6:**
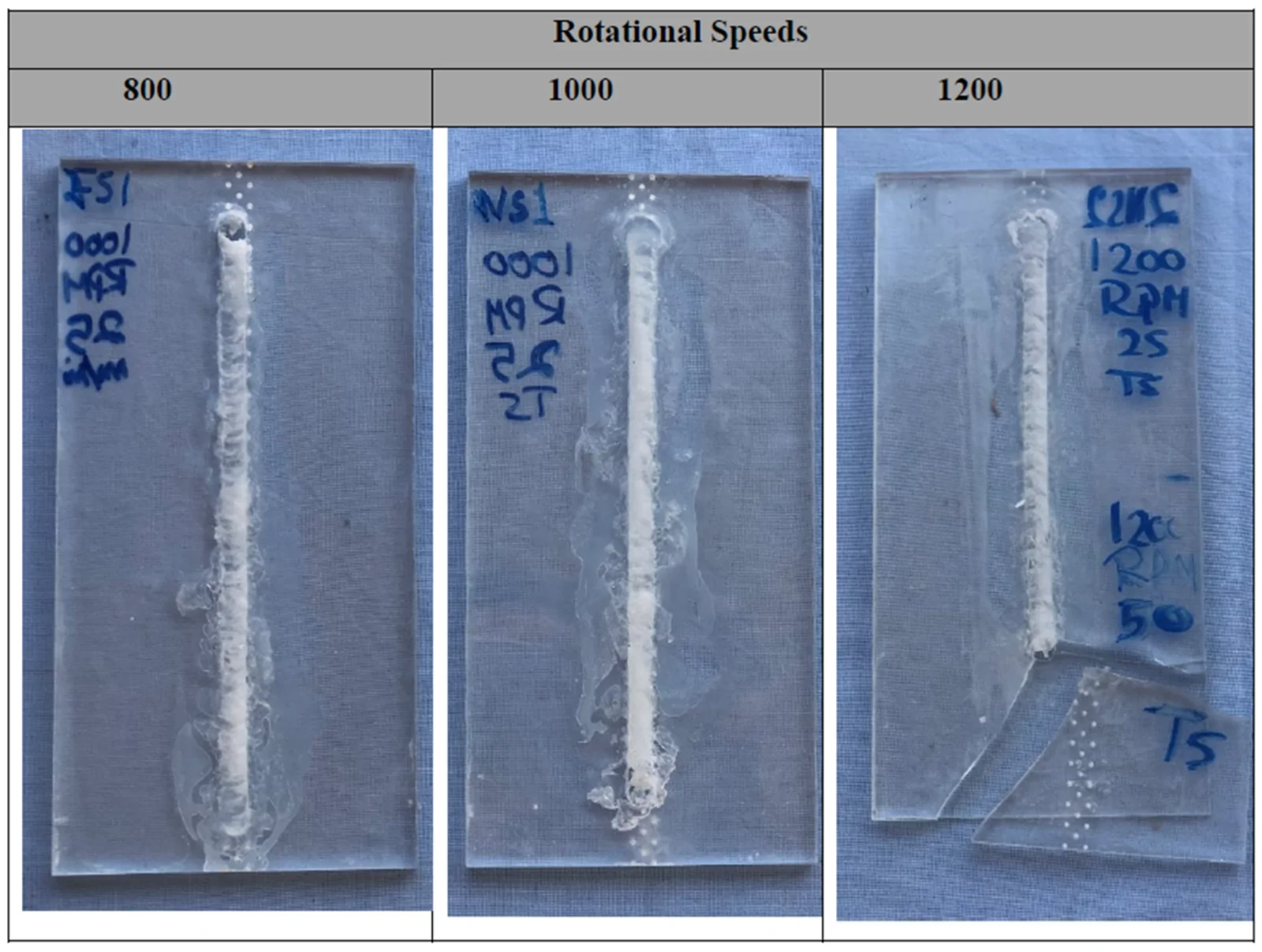
Optical images of the joint line in friction stir-processed samples.

To choose the optimum combination between low transverse speed and high rotational speed and to ensure even distribution while maintaining an adequate temperature to prevent overheating of the polymer, the difference between the fabricated composites’ processed line and cross section can be seen in Figure 6. At 800 rpm, the processed line exhibits alternating white and transparent regions, while at 1000 rpm it displays a uniformly white processed zone; the same is apparent in the cross section of both, which can be attributed to the fact that increasing the rotational speed enhances the mixing of the softened polymer and nanoparticles. Conversely, processing at 1200 rpm resulted in specimen fracture. Thermal monitoring during processing revealed that the combination of a 25 mm/min transverse speed and a 1000 rpm rotational speed consistently achieved the requisite polymer softening temperature range, justifying their selection as the optimal processing parameters, as this produced an average of 140 degrees surface temperature, whereas the plunge depth and tilt angle were 5 mm and 2°, respectively.

### 2.4. Material Characterizations

The friction stir processed (FSPed) samples were sectioned from the stir zone to visualize the material flow during FSP. After that, the cross-sections were mechanically polished with emery papers of different grades and observed using scanning electron microscopy (SEM), which was operated at an acceleration voltage of 20 kV using QUANTA FEG250. Morphological observations were carried out to reveal the dispersion state of nanoparticles in the composite matrix in different volume percentages and to evaluate the surface microstructure of the fabricated nanocomposite. Impact testing was performed in accordance with ASTM D256 [26], and the microhardness of the fabricated composite was tested using a Vickers microhardness tester with a 10 g load for 10 s, in accordance with ASTM E384 [27]. Finally, to determine the tensile strength, dog-bone-shaped specimens were cut from the center in a transverse direction of the processed line in accordance with ASTM D638 [28]; the test was performed using a servo-controlled universal testing machine at room temperature with a load cell of 400 KN with a 1 mm/min initial strain rate, and the average tensile strength was calculated. Finally, pin-on-disk wear was conducted on a tribometer. The disc is rotated at a selected speed. The elastic arm ensures a nearly fixed contact point and a stable specimen position on the disc. The wear rate of the pin is calculated from the weight of the material removed during the test. The speed was 1 m/s, the sliding distance was 450 m, and the load varied to 3, 6 and 9 N, and the average of weight loss and friction coefficient was obtained.

All mechanical tests were performed in triplicate (n=3), with data reported as the mean ± standard deviation (represented as error bars in all figures). The statistical significance of differences among groups was evaluated using One-Way Analysis of Variance (ANOVA) at a confidence level of α=0.05.

## 3. Results and Discussion

### 3.1. FSPed Zone Characterization

**Figure 7:**
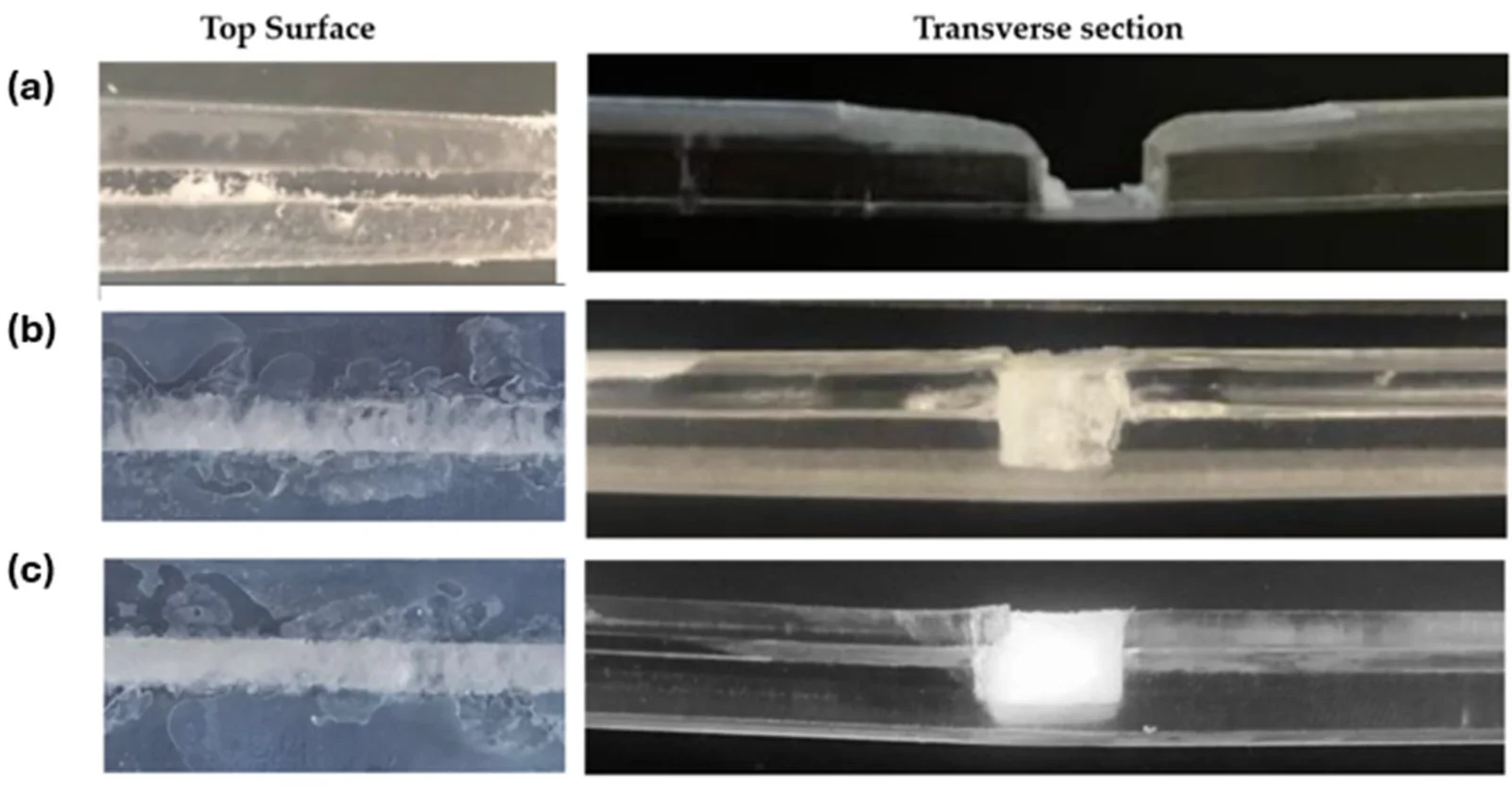
Optical images of the top surface and transverse cross section of the FSPed PMMA/Al_2_O_3_ nanocomposite (**a**) without surface lubrication, (**b**) with surface lubrication at 800 rpm and (**c**) with surface lubrication at 1000 rpm.

**Figure 8:**
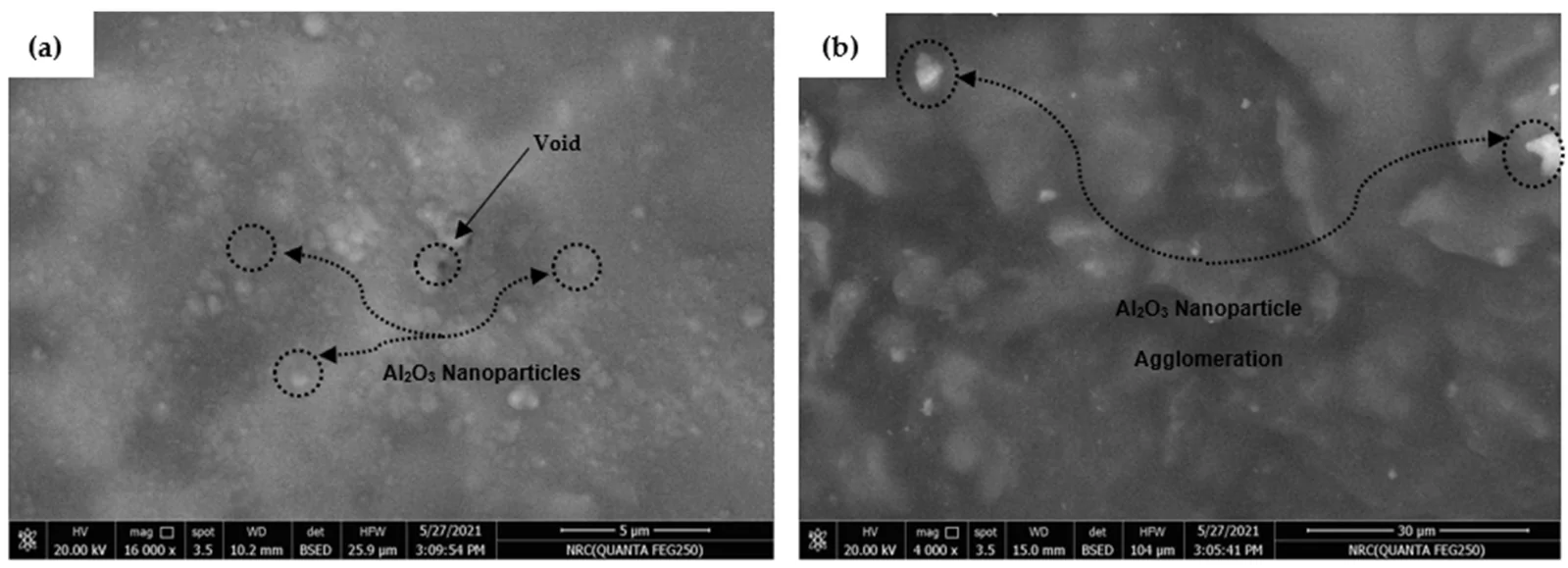
SEM image of nanocomposite cross-section containing (**a**) 15% vol and (**b**) 20% vol Al_2_O_3_.

During friction stir processing, the surface temperature of the nanocomposite was measured using a temperature gun, which indicated an average value of 140 °C. As a result, the polymer in both plates (2 mm and 4 mm) was softened, rearranged, and consolidated during processing, causing the formation of a white joint with an eddy current flow. It should be mentioned here that the glass transition temperature of the PMMA is quite low, about 110 °C [16], which indicates the experienced temperature during the FSP is even higher than Tg of this material, and this will have a significant implication on the PMMA after FSP; however, this implication is out of the scope of this study. The cross-section of the friction stir-processed PMMA without surface lubrication, shown in Figure 7a, shows a groove formed on the polymer due to direct contact between the FSP tool shoulder and the plate. On the other hand, using surface lubrication resulted in a sound FSPed zone with a symmetric stir zone with no voids or shrinkage bubbles, as can be observed from the transverse cross sections shown in Figure 7b (at 800 rpm) and Figure 7c (at 1000 rpm). The composite’s SEM images were taken using NRC (QUANTA FEG250) at different magnifications, as shown in Figure 8. The images show overall good dispersion and uniform distribution of nanoparticles inside the matrix, although some agglomerations are present. The relatively homogenous distribution of nanoparticles can be attributed to the configuration pattern used, where it is evident that the overall dispersion of Al_2_O_3_ was enhanced by using this method over the groove method used in previous literature, which reported the presence of agglomerations at lower Al_2_O_3_ volume percentages [6]. The SEM image of the 15% Al_2_O_3_ volume percentage showed the presence of a very small void, which can be attributed to the loss of volatile material during processing, while particle agglomeration can be seen in the 20% samples [6]. Observations suggest that the FSP of PMMA resulted in the formation of shrinkage holes, and as the concentration of nanoparticles increased, the nanoparticles were confined inside the shrinkage holes.

The stir zone shown is well distributed with no pin tip deformation, tool shoulder deformation, material overflow, or flashes. As the thermomechanical behavior is the main factor affecting the processed line formation and mechanical properties [29], the following observations can be attributed to the addition of a lubricating layer between the tool shoulder and polymer, which prevented shoulder deformation and overflow; the presence of the 2 mm thick plate, which successfully prevented scattering of the nanoparticles during processing or plate thickness reduction; and finally, the choice of transverse speed, rotational speed, and plunge depth, which ensured safe processing of the polymer to prohibit the formation of visual tunnels or pores.

Similar results were observed by previous work that utilized either a stationary shoulder attachment or submerged FSP, as both methods ensured sufficient material flow by containing the displaced material inside the nugget zone, which prevented material overflow, thickness reduction, and the formation of voids [30,31,32,33].

### 3.2. Hardness Results

**Figure 9:**
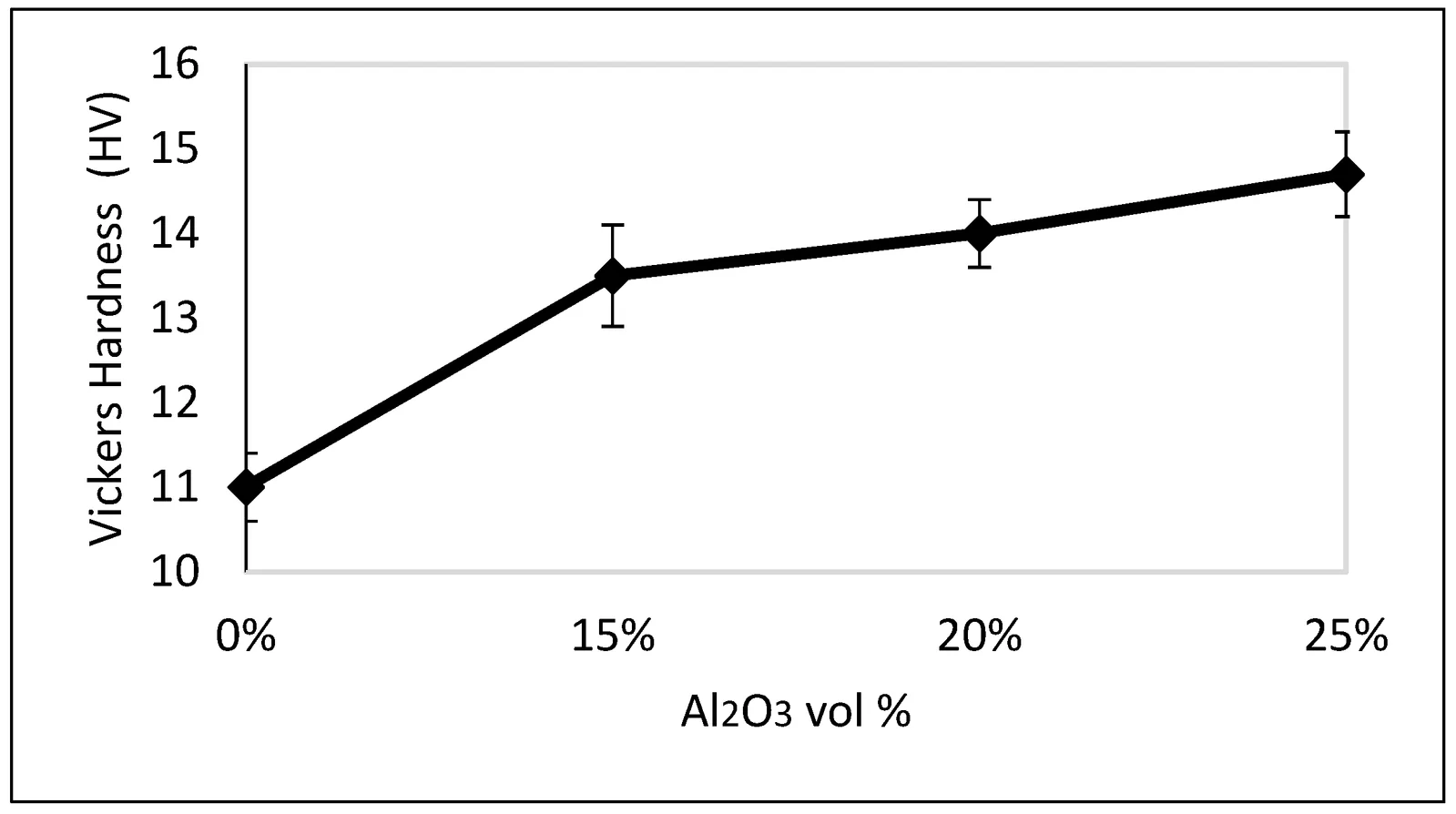
Effect of Al_2_O_3_ nanoparticles on the hardness of PMMA.

Figure 9 shows the microhardness results of the processed samples. The results show that microhardness increased with the addition of nano-alumina, with the hardness increasing up to 33% with the addition of 25% nano Al_2_O_3_ volume percentage. The improvement in PMMA hardness can be attributed to the addition of ceramic nanoparticles with good dispersion. The improvement in hardness is attributed to the addition of ceramic nanoparticles with relatively uniform dispersion. Similar results were observed in previous work, in which adding ceramic particles to the processed zone substantially improved the hardness of the composite nugget. This observation was attributed to the ceramic particles reducing the occurrence of voids, thus enhancing the overall quality of the processed composite [7,34].

### 3.3. Impact Energy Results

**Figure 10:**
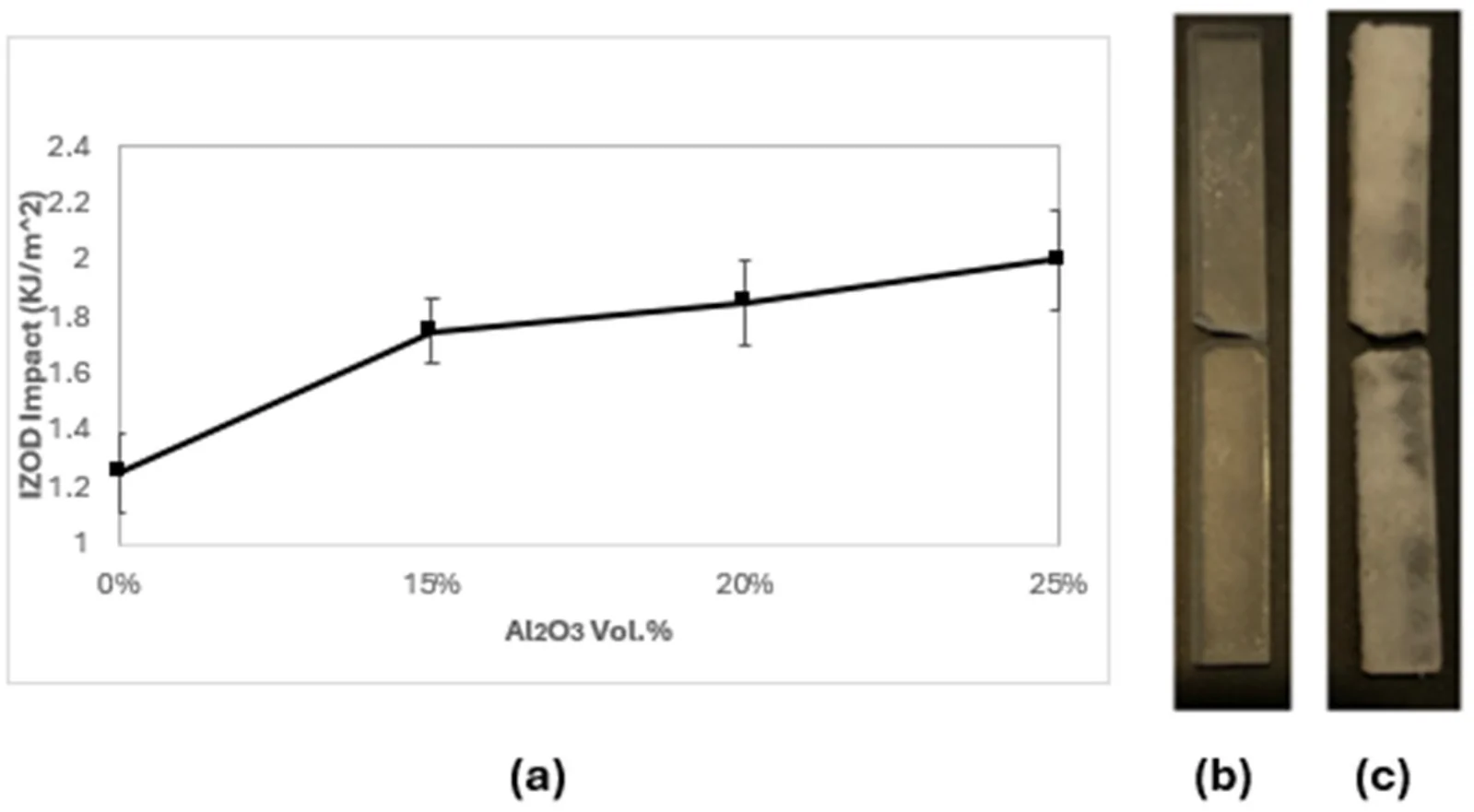
(**a**) Effect of Al_2_O_3_ nanoparticles on the impact strength of PMMA, fractured samples of (**b**) pure PMMA and (**c**) 25% vol Al_2_O_3_ nanocomposite.

To measure the effect of nano-alumina particles on the PMMA fracture energy, the IZOD impact test was performed. It was concluded that the addition of nanoparticles increased the impact energy absorbed, reaching an enhancement of up to 60% at 25 vol% of Al_2_O_3_, as shown in Figure 10. The increase in energy absorbed can be attributed to the strengthening effect of the alumina nanoparticles, which enhanced the fracture resistance of the polymer matrix composite, which has a high hardness, leading to an increase in the impact energy required to fracture the composite. Therefore, the presence of Al_2_O_3_ nanoparticles in the polymer matrix introduced deflection to crack growth due to their hardness and uniform distribution in the produced composite.

### 3.4. Tensile Test Results

**Figure 11:**
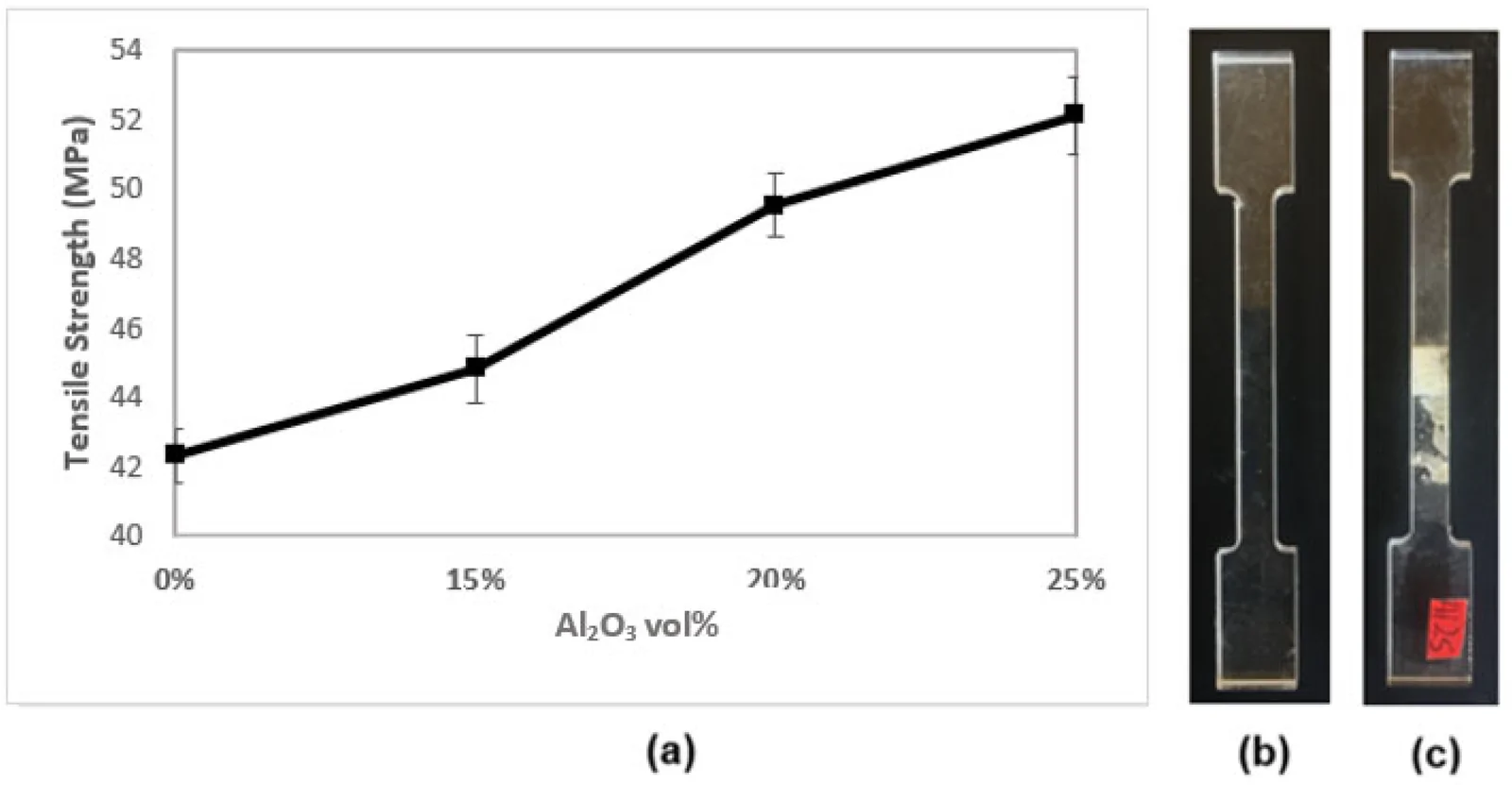
(**a**) Effect of Al_2_O_3_ nanoparticles on the tensile strength of PMMA, dog-bone-shaped specimen of (**b**) pure PMMA and (**c**) 25% vol Al_2_O_3_ nanocomposite.

As shown in Figure 11, increasing the concentration of Al_2_O_3_ nanoparticles increased the tensile strength of PMMA, whereas the tensile strength was enhanced by 23% at 25% vol concentration of nanoparticles. Despite the presence of some agglomerates, the overall particle dispersion and interfacial interaction provided an increase in strength, but agglomeration potentially limits further improvement [7,8,12]. Tensile strength is reportedly enhanced by ensuring that the temperature distribution throughout the material is uniform in the direction of thickness during processing [9]. This can be achieved through proper processing parameters. When the heat generated through friction is insufficient, the material does not flow adequately, and when there is excessive heat input, the plasticized materials overflow, causing defects to form, which in turn initiates cracks, ultimately reducing the material’s tensile strength.

### 3.5. Wear Test Results

**Figure 12:**
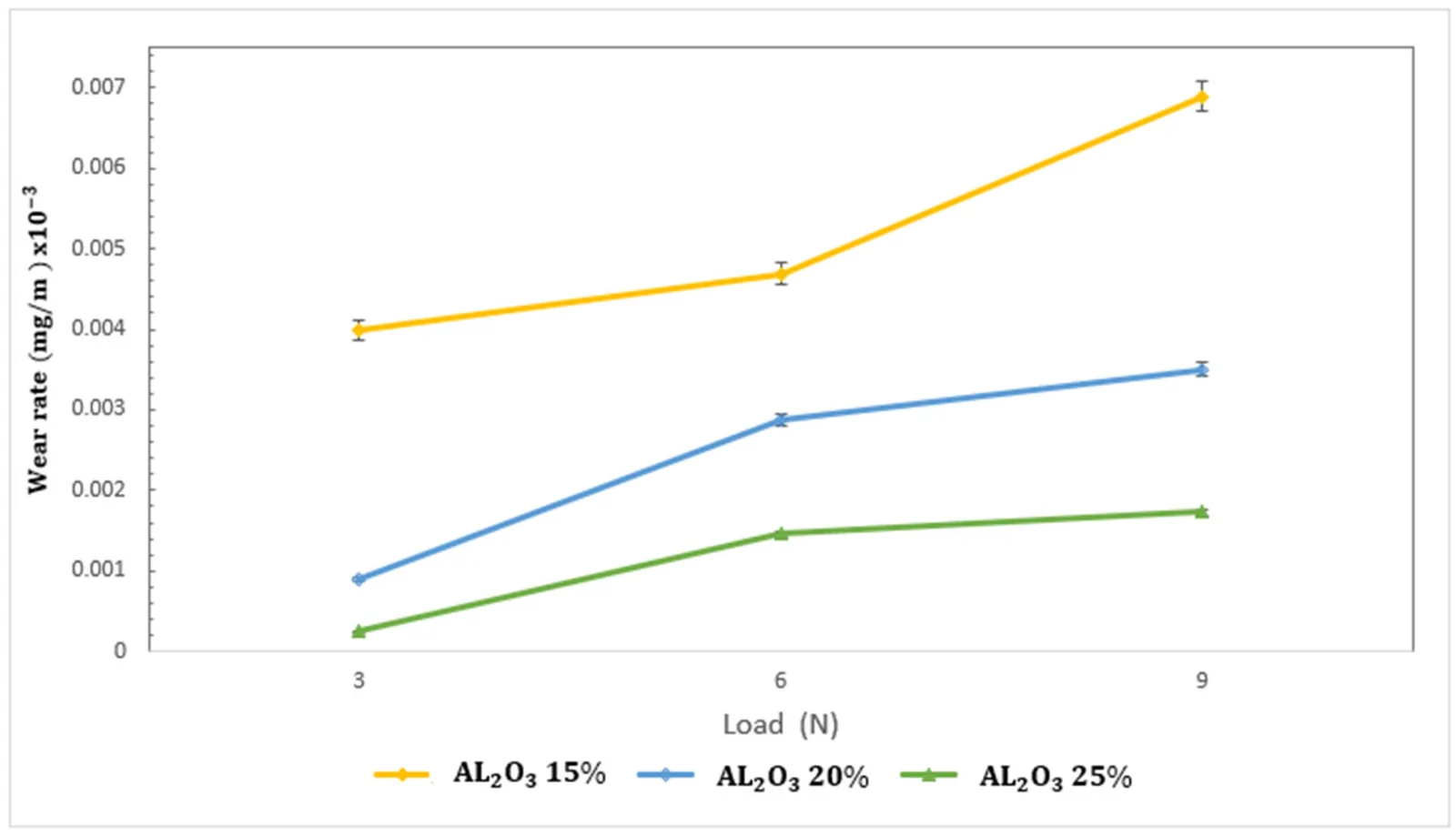
Plot of load versus wear rate for Al_2_O_3_ at different concentrations.

To calculate the wear rate, the mass loss of the polymer is divided by the sliding distance and expressed in units of mass loss per unit sliding distance. The results have been compiled and graphically represented in Figure 12. The analysis of the data indicates that the incorporation of hard nanoparticles led to a decrease in the average weight loss of the nanocomposites. Additionally, the wear rate was observed to increase with higher applied loads. Interestingly, the results also showed that as the concentration of nanoparticles was increased, there was a corresponding reduction in the amount of worn mass. While Al_2_O_3_ nanoparticles are known to be abrasive, the addition of these nanoparticles has been shown to improve the wear resistance of polymeric base nanocomposites. In correlation to the hardness results obtained in Figure 9, the addition of nanoparticles led to an increase in hardness, which can result in improved resistance to wear. This occurs because the nanoparticles act as barriers to the propagation of cracks and other forms of damage, preventing the material from being worn away.

**Figure 13:**
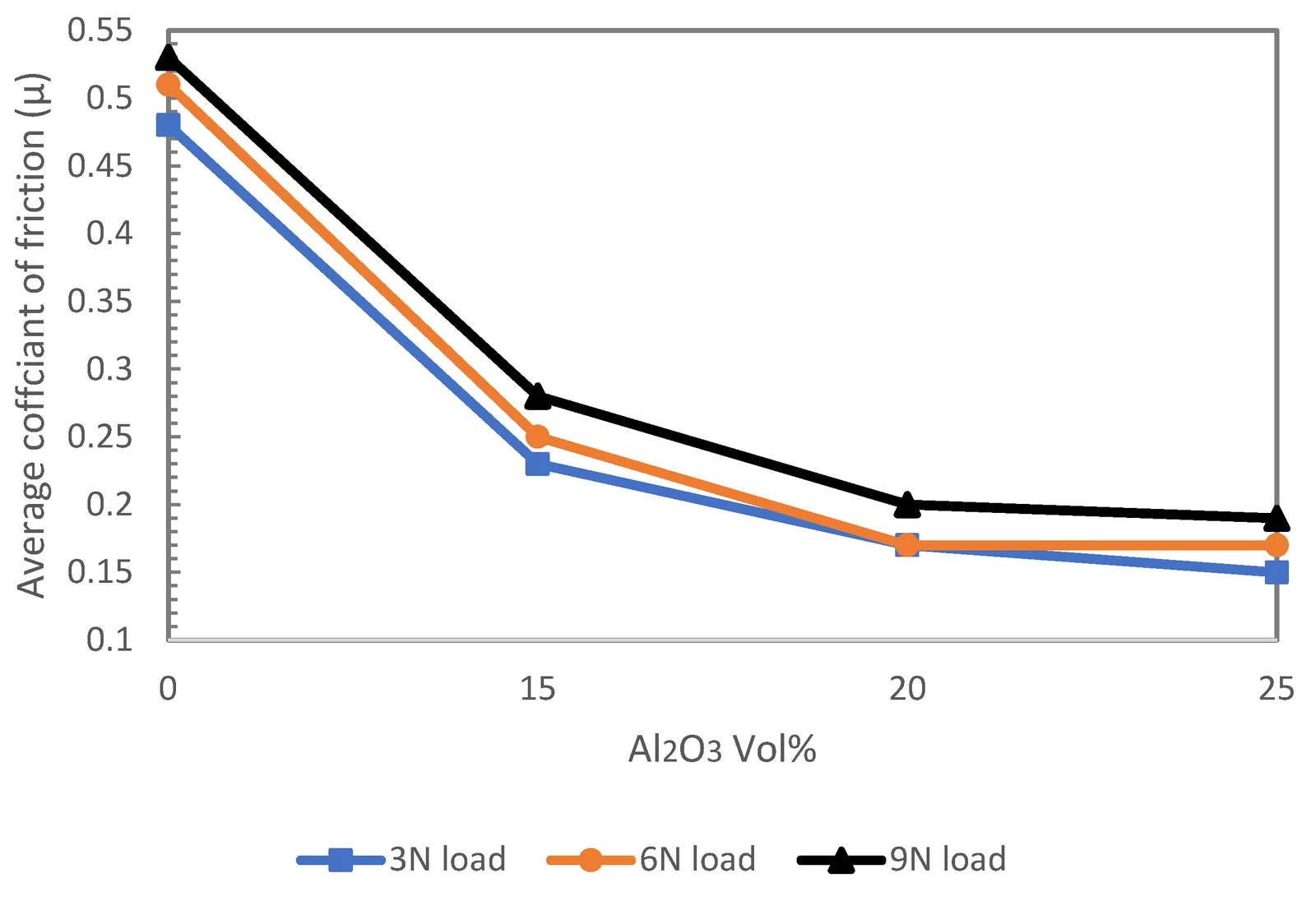
Effect of nanoparticles on average coefficient of friction of PMMA at different loads.

Figure 13 illustrates the composites’ average coefficient of friction at a 450 m sliding distance. The unfilled sample is reported to provide the highest friction coefficient value under various applied loads. By analyzing the data obtained, it can be shown that the incorporation of Al_2_O_3_ nanoparticles significantly reduces friction coefficient values, whereas PMMA frictional coefficient values tend to decrease as nanoparticle concentration rises. It can be inferred that the surface roughness of the nanocomposite films affects their frictional behaviour. These results can be attributed to the ability of nanoparticles to generate a self-lubricating layer on the contact region and function as modifiers, which improved friction performance and helped to minimize friction between rubbing surfaces.

### 3.6. General Discussion

Friction stir processing as a solid-state process [35] has been used to develop PMMA/Al_2_O_3_ nanocomposites where several FSP parameters have been investigated, and the developed nanocomposites were characterized in terms of surface appearance, microstructure, hardness, and tensile and impact toughness. The methodology developed by Ahmed et al. [34] for the use of surface lubrication on the top surface of a polymer to prevent the wear of the top surface and prevent groove formation [36] on the top surface, causing sheet thinning, has been applied in this work. This methodology resulted in a sound FSP zone for the whole thickness without thinning, and thus, the mechanical properties were improved in terms of hardness and tensile strength. Hardness was improved from 11 Hv for the PMMA sheets to about 15 Hv for the nanocomposite with 25% alumina. Tensile strength was increased from 42 MPa for the PMMA sheet to about 53 MPa for the 25% alumina nanocomposite. Interestingly, the impact toughness was also improved from 1.2 to about 2 KJ/$m^{2}$ for the 25% alumina nanocomposite. These improvements can be attributed to the enhancement of the load-carrying ability of the polymer matrix due to the addition of alumina nanoparticles [37,38,39,40]. Furthermore, nanoparticle distribution and dispersion were very good in the polymer matrix due to the use of a square grid of holes and a top surface sheet to prevent nanoparticle escape during stirring.

## 4. Conclusions

In this study, friction stir processing was used to fabricate a PMMA/Al_2_O_3_ nanocomposite. The microstructure and mechanical properties of the produced composite with nano-alumina particles ranging from 15 to 25 vol% were investigated. From the results, the following findings can be summarized:

1. During processing, increasing the rotational speed causes material degradation, as the polymer has low thermal conductivity. However, decreasing the rotational speed causes insufficient mixing between polymer and particles.
2. The rotational speed, transverse speed, and tilt angle should be varied to reach the optimum processing temperature for the polymer used to prevent processing defects, such as voids, agglomerations, uneven melting, and crack formation.
3. The addition of a lubricant during FSP is a promising, simple, and low-cost technique that eliminates thickness reduction and wear produced due to the dry contact between the shoulder and plates.
4. Using adequate processing parameters of 1000 rpm and 25 mm/min transverse speed, a homogeneous, uniform composite surface layer was produced with no macro defects in the cross-section.
5. The suggested configuration of holes led to a relatively uniform distribution of nanoparticles and reduced agglomerations.
6. Through wear tests, it was observed that an increase in load exhibited a linear correlation with weight loss, which indicates a steady dry sliding behaviour. The incorporation of nanoparticles demonstrated a reduction in both the friction coefficient and weight loss. Consequently, the presence of the hard inclusions not only enhanced the strength and hardness of PMMA but also served as a solid lubricant to lower the friction coefficient in the sliding process.
7. The addition of alumina nanoparticles to PMMA increased the microhardness and impact energy of the polymer, whereas the nanocomposite of 25 vol% Al_2_O_3_ nanoparticles had a hardness of 14.7 HV, impact energy of 2 KJ/m^2^, and tensile strength of 52.1 MPa.

## Figures and Tables

**Table 1:** Calculated hole diameters for different volume concentrations of the nanoparticle.

Al_2_O_3_ Volume Percentage	Hole Diameter (mm)
15%	1.77
20%	2.04
25%	2.28

**Table 2:** Generic data from previous literature (NM accounts for not mentioned).

No.	Ref. No	Base Material	FSP Methodology				
			Tool Design		Processing Parameter		
			Material	Shape and Dimension	Rotation Speed (RPM)	Transverse Speed (mm/min)	Angle of Tilt (Degree)
1	[7]	PMMA	High-speed tool steel	Frustum Diameter: 4–6 mm	CCW 1600 RPM	25	1
2	[8]	Polypropylene	H13 Hot worked steel	Diameter: 9 and length 6 mm	1000	50	NM
3	[9]	Polyamide	NM	Diameter: 9 and length 6 mm	2000	100	NM
4	[12]	HDPE	H13 Steel	Conical screwed diameter of 8–10 mm and length 6 mm	1800	30	NM
5	[10]	HDPE	H13 Steel	Square 20 × 5 × 2 mm	900	160	1

**Table 3:** FSP parameters were investigated in the preliminary tests.

Tool Design		Processing Parameter			
Material	Shape	Rotation Speed (RPM)	Transverse Speed (mm/min)	Angle of Tilt (Degrees)	Plunge Depth (mm)
High-speed tool steel (H13)	Cylindrical	800, 1000, 1200	25, 50	1°, 2°	4

## Data Availability

This will be available upon request through the corresponding author.
